# Impact of dialysis modality on survival after kidney transplant failure: A systematic review and meta-analysis

**DOI:** 10.12669/pjms.41.6.12079

**Published:** 2025-06

**Authors:** Na Zhu, Li Mei

**Affiliations:** 1 Na Zhu Department of Nephrology, Ningbo Medical Center Lihuili Hospital, Ningbo, Zhejiang Province 315040, P.R. China; 2 Li Mei Department of ICU, Ningbo Medical Center Lihuili Hospital, Ningbo, Zhejiang Province 315040, P.R. China

**Keywords:** Allograft failure, Hemodialysis, Kidney transplant, Mortality, Peritoneal dialysis

## Abstract

**Objective:**

We reviewed evidence on survival of dialysis after graft failure (DAGF) patients based on the selected dialysis modality (peritoneal dialysis [PD] vs hemodialysis[HD])

**Methods:**

PubMed, CENTRAL, Embase, Scopus, and Web of Science were searched for all type of studies comparing PD with HD in DAGF patients and reporting survival rates, technique survival or complications. The last date of the search was May 5, 2024. The Newcastle-Ottawa Scale was used to assess study quality. We extracted both crude and multivariable-adjusted data from the studies for the meta-analysis which were pooled as odds ratio (OR) and Hazard ratios (HR) respectively. Outcomes were assessed at the longest follow-up of the included studies.

**Results:**

Seven studies were included comparing 2494 patients on PD and 4041 patients on HD. Meta-analysis of crude data showed that mortality rates did not differ between PD and HD in patients receiving DAGF (OR: 0.98 95% CI: 0.76, 1.27 I^2^=50%). Meta-analysis of adjusted data also showed that the dialysis modality (PD or HD) had no impact on survival rates in patients receiving DAGF (HR: 0.93 95% CI: 0.73, 1.18 I^2^=50%). Sensitivity analysis did not change the significance of the results. Data was limited for other outcomes.

**Conclusions:**

Very-:low quality evidence mostly from retrospective studies shows that dialysis modality may not impact survival rates in patients returning to DAGF. There is a need for robust randomized controlled trials with large sample sizes to provide better evidence.

## INTRODUCTION

Chronic kidney disease (CKD) continues to be a major public healthcare problem affecting about 700 million individuals worldwide.^1^ CKD frequently progresses to end-stage kidney disease (ESKD) with complete loss of renal function requiring renal replacement therapy or kidney transplant for survival.^2^ With increased social awareness, improvements in therapeutics, and surgical strategies, the incidence of kidney transplantation has grown significantly. However, both short-term and long-term outcomes have not improved proportionately resulting in increased loss of allografts.^3^ Contemporary data shows that graft failure rates can range from 10.6- 31.5%^4^,^5^ resulting in a large proportion of patients requiring dialysis after graft failure.(DAGF) Indeed, graft loss is now the fourth most common cause of incident dialysis.^6^

Peritoneal dialysis (PD) and hemodialysis (HD) are the two commonly used dialysis for ESKD. However, choosing one over the other, especially in a cohort of failed allografts is a daunting task given that there are no clear guidelines on dialysis management of failed allografts.^7^,^8^ Patients requiring DAGF are significantly different from transplant-naïve candidates as they have been subjected to immunosuppression, have a residual non-functional or minimally functional allograft, and present with a chronic inflammatory state.^9^ In this context, does the choice of dialysis modality alters patient survival is unclear. For the general CKD population, PD has shown early survival benefits as compared to HD despite showing no long-term differences in mortality.^10^ However, is this replicated in patients returning to DAGF? To answer this clinical query, we conducted this first systematic review and meta-analysis in the literature comparing survival in patients returning to DAGF based on the choice of dialysis modality i.e. PD vs HD.

## METHODS

This study follows the PRISMA^11^ reporting guidelines. We registered the protocol of the review on PROSPERO with the number CRD42024541074. Two reviewers looked for human studies on the electronic databases of PubMed, CENTRAL, Embase, Scopus, and Web of Science from the earliest possible date to 5^th^ May 2024. No language restriction was applied. The reviewers used both MeSH and free keywords to identify relevant studies. We used Boolean operators and devised the following search query which was replicated across databases: (transplant) OR (transplantation) OR (allograft)) AND ((renal) OR (kidney) AND (peritoneal dialysis) AND (hemodialysis) AND ((failed) OR (failure) OR (loss)) (Supplementary Table-I). Further, references of included publications were manually scanned to identify the possible relevant articles.

**Supplementary Table-I:** Search strategy of all databases.

Database	Strategy
Embase	1. ’ transplant’ OR ‘transplantation’ OR ‘allograft’ 2. ‘renal’ OR ‘kidney’ 3. ’peritoneal dialysis’ AND ‘hemodialysis’ 4. ‘failed’ OR ‘failure’ OR ‘loss’ 5. #1 AND #2 AND #3 AND #4
PubMed	(((((transplant) OR (transplantation)) OR (allograft)) AND ((renal) OR (kidney))) AND ((peritoneal dialysis) AND (hemodialysis))) AND (((failed) OR (failure)) OR (loss))
Scopus	(TITLE-ABS-KEY-AUTH (transplant OR transplantation OR allograft)) AND (TITLE-ABS-KEY-AUTH (renal OR kidney)) AND (TITLE-ABS-KEY-AUTH (peritoneal dialysis) AND hemodialysis)) AND (TITLE-ABS-KEY-AUTH (failed OR failure OR loss))
Web of Science	(((((transplant) OR (transplantation)) OR (allograft)) AND ((renal) OR (kidney))) AND ((peritoneal dialysis) AND (hemodialysis))) AND (((failed) OR (failure)) OR (loss))
CENTRAL	(transplant: ti,ab,kw OR transplantation: ti,ab,kw OR allograft: ti,ab,kw) AND (renal ti,ab,kw OR kidney) ti,ab,kw AND (peritoneal dialysis: ti,ab,kw AND hemodialysis ti,ab,kw) AND (failed ti,ab,kw OR failure ti,ab,kw OR loss ti,ab,kw)

Retrieved articles from all databases were combined and duplications were removed. Primary screening involved the elimination of non-relevant studies by reading the titles and abstracts. Important studies were identified and their full-texts retrieved. These underwent secondary screening by examination of full texts. Disagreements were discussed by discussion amongst the two reviewers.

### Inclusion criteria:

- All comparative study designs
- Conducted on subjects requiring DAGF
- Comparing outcomes between PD and HD
- Reporting crude or adjusted survival rates or any other outcomes like technique survival and complications.

### Exclusion Criteria:

- Studies not comparing outcomes based on dialysis modality
- Editorials, case series, case reports, and duplicate studies were excluded.

### Primary and secondary outcomes:

The primary aim was to assess survival between PD and HD patients. Secondary outcomes were kept open-ended and included all outcomes reported by the included studies.

### Data and risk of bias:

The reviewers formulated a table pre-defined form was used to collect information on author name, year of publication, location, study design, inclusion/exclusion criteria, sample size, demographic details, comorbidities, dialysis modality before transplant, pre-transplant dialysis duration, length of allograft function, switch in modality in DAGF, follow-up, and outcomes reported.

The authors chose to use the Newcastle-Ottawa Scale (NOS) to assess study quality.^12^ Every study was assessed for selection of cohort, comparability of the study and control groups, outcome measurement, and availability of follow-up and awarded stars ranging from zero to nine. Lower scores indicated low while higher scores meant high quality. Two authors conducted the bias assessment and any differences of opinion were resolved by discussion.

### Statistical analysis:

All analyses were conducted in “Review Manager” (RevMan, version 5.3; Denmark; 2014). We extracted both crude and multivariable-adjusted data from the studies for the meta-analysis. For crude mortality data, the longest follow-up reported by the studies was used. Such data was combined to generate the odds ratio (OR) and 95% confidence intervals (CI) as the pooled outcome. Adjusted data was further pooled by using the generic inverse variance function of the Review Manager and Hazard ratios (HR) were calculated. Significant heterogeneity was defined by the I^2^ statistics ≥50% and/or p < 0.10 of the Cochrane Q test. We chose a random effect model for all meta-analyses. Funnel plots were used to examine publication bias. To investigate the impact of individual studies on the overall risk summary, we performed a sensitivity analysis by removing one study at a time. Significance levels were set at 5%. Certainty of evidence was examined via GRADE method.

## RESULTS

**Fig.1:**
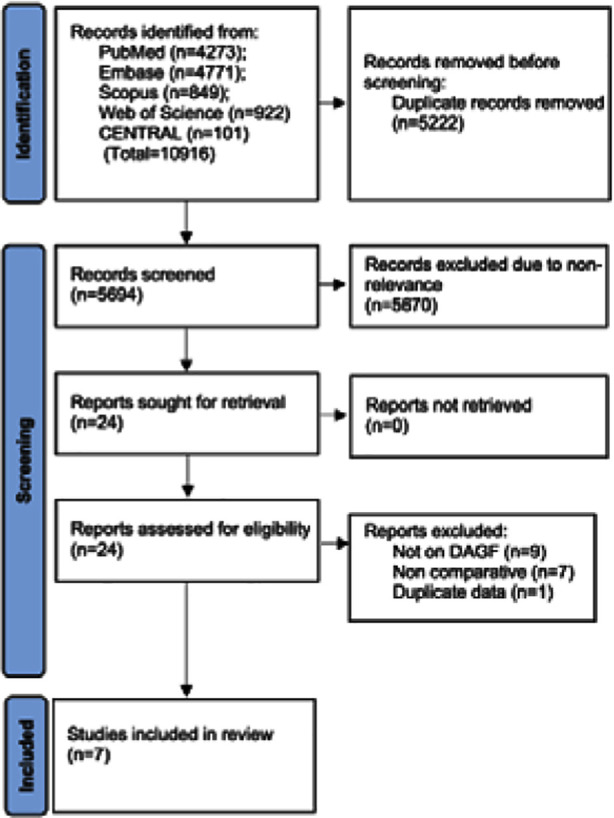
PRISMA Flowchart.

The flowchart of study selection is shown in Fig.1. Twenty four studies were selected for full-text analysis of which seven^13^–^19^ were selected for the review. There was complete agreement between the reviewers for the selection of all studies. Only one study of Davies et al^18^ was a prospective cohort study while the remaining were retrospective cohort studies.

Three studies included only adult populations, one study included only pediatric populations and the remaining studies included all patients (Table-I). The seven studies included 2494 patients with PD and 4041 patients with HD. One study used propensity score matching where 1865 of 14248 HD patients were propensity score matched with another 1865 PD patients. Crude mortality rates were available for the 14248 HD patients while adjusted data was available for the propensity score matched population. Two studies reported pre-transplant dialysis duration with no statistically significant difference between the two groups. The length of allograft function was not significantly different between PD and HD groups in most studies. Only three studies reported a switch in modality following DAGF. A higher percentage of PD patients switched in all three studies. Follow-up duration differed significantly between studies. The NOS scores of the studies varied from six to eight (Supplementary Table-II).

**Table-I:** Details of included studies.

Study	Inclusion/ exclusion criteria Groups: PD and HD	Sample size	Male gender	Age (years)	DM (%)	Cardiac disease (%)	PD pre-transplant (%)	Pre-transplant dialysis duration (months)	Length of allograft function (months)	Switch in modality (%)	Adjusted variables	Follow-up
La Porta 2021^13^ Italy	<18 years undergoing DAGF Excluded: Patients who had access to the first transplant as pre-emptive	41 77	56.1 63.6	11.5 14.6	NR	NR	90.2 48.1	17.8 [8.7-31.6] 22.5 [12.8-33.7]	NR	17 5	Gender, age, primary kidney disease, pre-transplant dialysis, dialysis cycle, comorbidities, pre-transplant dialysis duration	6 months-18 years
Catalan 2021^16^ Spain	All patients undergoing DAGF Excluded: transplant function <3 months	86 89	45 51	NR	13 8	28 63	NR	NR	NR	31.4 1.1	Age, vascular comorbidity, suboptimal access use	24 months
Perl 2013^15^ USA	Adult patients undergoing DAGF Excluded: underwent repeat transplant	1865 1865*	49.1 51.8	≥60: 49.1% ≥60: 51.8%	NR	23.9 25.6	NR	NR	<1 year: 35.2% <1 year: 34%	NR	Age, race, sex, body mass index, serum albumin, history of smoking, history of substance abuse, cause of initial end-stage renal disease, comorbidities, employment status, duration of graft function, pretransplant dialysis duration, estimated glomerular filtration rate at dialysis initiation	2 years
Perl 2011^14^ Canada	Adult patients undergoing DAGF Excluded: Pre-1988 patients, underwent repeat transplant	389 1721	47 64.2	44.1 47.4	22.9 18	10.5 10.5	55.3 15.1	18.3 [9.1-33.9] 14.8 [7.6-24.8]	<1 year: 37.3% <1 year: 31.8%	NR	Age, sex, ethnicity, dialysis year, etiology, comorbidities, length of allograft function, pretransplant dialysis, living donor	3 years
Chung 2011^19^ Korea	Adult patients undergoing DAGF Excluded: survived <3 months, repeat transplant	64 233	62.5 72.5	41.8 41.3	21.9 23.2	7.8 4.7	37.7 10.4	21.4± 25.7 17.5± 21.1	83.3± 64 74.9± 63	NR	Gender, graft survival duration, transplant nephrectomy, donor type, HLA mismatch number, hemoglobin, and serum creatinine level	Up to 10 years
de Jonge 2006^17^ Belgium	All patients undergoing DAGF Excluded: NR	21 39	42.9 61.8	46.3 48.9	19 25.6	9.5 25.6	NR	NR	144 [26.8-175.7] 59.1 [9-131.1]	2 0	NR	NR
Davies 2001^18^ UK	All patients undergoing DAGF Excluded: transplant function <6 months	28 17	NR	41.2 38.9	18 0	14 35	NR	NR	NR	NR	NR	Up to 12 years

DM, diabetes mellitus; DAGF, dialysis after graft failure; HD, hemodialysis; PD, peritoneal dialysis; NR, not reported

* total HD sample size was 14248 out of which 1865 were propensity score matched with PD patient.

**Supplementary Table-II:** Risk of Bias in included studies

Study	Selection of cohort	Comparability of groups	Outcome assessment	Total NOS score
La Porta 2021^13^ (Italy)	****	**	**	8
Catalan 2021^16^ (Spain)	****	**	**	8
Perl 2013^15^ (USA)	****	*	**	7
Perl 2011^14^ (Canada)	****	**	**	8
Chung 2011^19^ (Korea)	****	**	**	8
de Jonge 2006^17^ (Belgium)	****	-	**	6
Davies 2001^18^ (UK)	****	-	**	6

All studies reported on survival post-DAGF (Supplementary Table-II). De Jonge et al.^17^ noted that the number of patients dying in the HD group was significantly higher than in the PD group with one death every 82.9 patient months in the HD group and only one death every 132.6 patient months in the PD group. However, the survival curves showed no difference between the two groups. For the remaining studies, data showed that survival did not differ between PD and HD groups.

Only one study reported complication rates. La Porta et al.^13^ found a significantly higher number of clinical and dialysis-related complications in the PD group as compared to the HD group. On the other hand, hospitalization rates were significantly lower in the PD group as compared to the HD group in the study of Catalan et al.^16^ Two studies reported retransplantation rates. Perl et al.^15^ found no difference in replantation rates between the two groups while de Jonge et al.^17^ noted that retransplantation rates were higher in the PD group.

**Fig.2:**
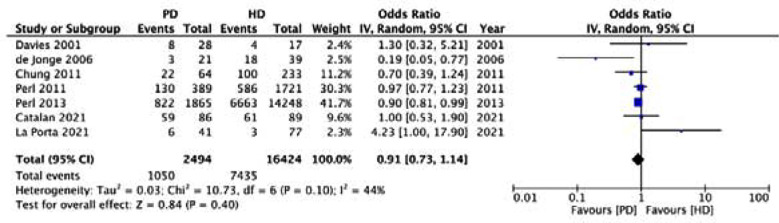
Meta-analysis of crude mortality rates between PD and HD groups in DAGF patients.

**Supplementary Fig.1:**
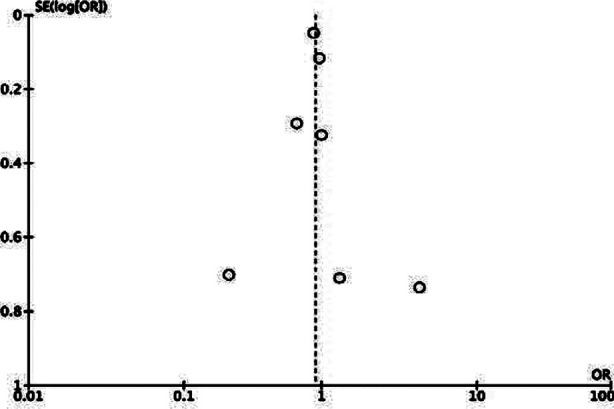
Funnel plot for publication bias.

Meta-analysis of crude data showed that mortality rates did not differ between PD and HD in DAGF (OR: 0.91 95% CI: 0.73, 1.14 I^2^=44%) (Fig.2). We could not find any evidence of publication bias from the funnel plot (Supplementary Fig.1). Sensitivity analysis results are shown in Supplementary Table-III. As noted in the table, the OR varied from 0.88 to 0.94 on the sequential exclusion of individual studies and the results remained statistically non-significant.

**Supplementary Table-III:** Outcomes reported by the included studies.

Study	Outcome	Results
La Porta 2021	Survival	At the end of follow-up, mortality in PD (13.6%) was higher than HD (4.2%). After adjustment of covariates, there was no difference in survival between PD and HD (HR 2.15; 95% CI 0.54, 8.6)
	Complications	104 complications noted in 41 PD patients (1 episode/17.4 months) while 90 complications noted in 77 HD patients (1 episode/31.4 months). Patients on PD had a higher rate of both clinical and dialysis-related complications as compared with those on HD.
Catalan 2021	Survival	No difference in survival between PD and HD at 1 year (95% vs 92%) and a 5 years (69% vs 69%) respectively. After adjusting for age, vascular comorbidity, non-optimal use of access, PD patients had better overall survival (HR: 0.36 95% CI: 0.148, 0.894)
	Hospitalization	After adjusting for age, vascular comorbidity, non-optimal use of access, eGFR at beginning of dialysis, PD resulted in lower risk of hospitalization (HR: 0.52 95% CI: 0.369, 0.743)
	Graft intolerance	After adjusting for age, eGFR at beginning of dialysis, PD was associated with reduced graft intolerance symptoms (HR: 0.307 95% CI: 0.142, 0.758)
Perl 2013	Survival	During the study period, mortality in PD group was 44% and in the HD group was 47%. After adjustment for age, race, sex, body mass index, serum albumin, history of smoking, history of substance abuse, cause of initial end-stage renal disease, comorbidities, employment status, duration of graft function, pretransplant dialysis duration, eGFR at dialysis initiation, survival within the first year was greater for PD patients (HR: 0.83; 95% CI: 0.69, 0.99) but at the end of follow-up no difference was noted in survival (HR: 1.03; 95% CI: 0.93, 1.14)
	Retransplantation	No significant difference between PD and HD for retransplantation rates (HR: 1.02 95% CI: 0.93, 1.12)
Perl 2011	Survival	No difference in survival between PD and HD (HR: 0.95 95% CI: 0.76, 1.18). No impact of dialysis modality on survival within 2 years and after 2 years of graft failure.
Chung 2011	Survival	The survival rates of PD patients at 1, 5, and 10 years were 98%, 85%, and 65% respectively, and those on HD were 89%, 70%, and 57% respectively with no statistical significant difference. Adjusted analysis also showed no difference is survival between the two modalities (HR: 0.88 95% CI: 0.49, 1.49)
de Jonge 2006	Survival	Number of patients dying in the HD group were significantly higher than in the PD group. There was one death every 82.9 patient months in the HD group and only one death every 132.6 patient months in the PD group. Kaplan–Meier survival curves did not differ significantly between the two groups. Cause of deaths did not differ between the two groups.
	Retransplantation	Number of patients receiving retransplantation were significantly higher in PD compared to HD groups (38.1 vs 20.5%)
Davies 2001	Survival	No impact of dialysis modality on survival after graft failure but median survival was longer in the PD group.

**Fig.3:**
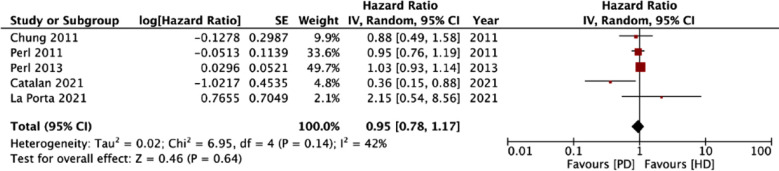
Meta-analysis of adjusted survival rates between PD and HD groups in DAGF patients.

Five studies reported adjusted survival data. Pooled analysis showed that dialysis modality (PD vs HD) had no impact on survival in patients receiving DAGF (HR: 0.95 95% CI: 0.78, 1.17 I^2^=42%) (Fig.3). On sensitivity analysis, the HR varied from 0.85 to 1.02 and the effect size remained non-significant (Supplementary Table-IV). On GRADE assessment, the certainty of evidence was “very low” for both crude and adjusted mortality outcomes (Supplementary Table-V).

**Supplementary Table-IV:** Sensitivity analysis for the meta-analyses.

Study removed	Odds ratio [95% confidence intervals]	I^2^
***Crude mortality***		
Davies 2001	0.90 [0.71, 1.14]	52
de Jonge 2006	0.92 [0.80, 1.06]	15
Perl 2011	0.88 [0.59, 1.32]	52
Perl 2013	0.91 [0.59, 1.40]	53
Chung 2011	0.94 [0.73, 1.21]	50
Catalan 2021	0.90 [0.69, 1.16]	53
La Porta 2021	0.89 [0.77, 1.04]	21
***Adjusted survival***	***Hazard ratio [95% confidence intervals]***	
Chung 2011	0.95 [0.75, 1.21]	56
Perl 2011	0.88 [0.57, 1.38]	55
Perl 2013	0.85 [0.55, 1.31]	48
Catalan 2021	1.02 [0.93, 1.11]	0
La Porta 2021	0.94 [0.77, 1.15]	48

**Supplementary Table-V:** GRADE assessment of evidence.

	Crude mortality	Adjusted mortality
Number of studies	7	5
***Downgrade quality of evidence***		
Risk of bias	Very serious*	Serious^
Inconsistency	No	No
Indirectness	No	No
Imprecision	No	No
***Publication bias***		
***Upgrade quality of evidence***		
Large effect	No	No
Plausible confounding	No	No
Dose-response	No	No
Overall certainty of Evidence	Very low	Very low

* No matching of baseline characteristics between the study groups in any study, ^NOS score ranged from 7-8 in the included studies

## DISCUSSION

To the best of our knowledge, our study is the first meta-analysis in the literature examining the role of dialysis modality in predicting survival in patients returning to DAGF. Results showed that there seems to be no difference in survival rates between PD and HD in DAGF patients. Results were robust and demonstrated no change in sensitivity analysis.

Kidney transplantation failure remains one of the most difficult transitions for CKD patients. Management of such patients involves treating CKD complications, planning and restarting dialysis, immunosuppression withdrawal, allograft nephrectomy, and further evaluation for a potential retransplantation.^20^ Research shows that patients with failing kidney allografts are more morbid than nontransplant controls with a similar degree of CKD^21^ Further, patients returning to dialysis often have a chronic inflammatory state.^9^ Immunosuppressive therapy increases the risk of nephrotoxicity and infections in such patients which along with metabolic alterations can increase the risk of mortality.^22^ Given the complex nature of the disease, it is not surprising to note that transplant failure patients have a 1.5 times higher risk of mortality as compared to matched CKD patients who never received a transplant.^21^ A systematic review^23^ has shown that in DAGF patients, the risk of mortality is highest in the first year of return to dialysis (about 12%) and reduces in the next 2-4 years (5-6%).

However, does this risk vary with the type of dialysis modality? It is well known that PD offers early survival benefits as compared to HD and with longer follow-up this difference evens out resulting in no statistically significant difference in survival.^10^ However, to generalize these results to the entire spectrum of CKD patients would be improper. In special cohorts, there have been differences in survival based on the choice of dialysis modality. A meta-analysis has shown that PD results in a 17% greater risk of mortality as compared to HD.^24^ On the other hand, in urgent-start cases, HD is associated with a higher risk of mortality as compared to PD.^25^ In obese and diabetic patients, evidence is unclear as to which modality results in better outcomes.^26^

In this review, we examined the impact of dialysis modality on the survival outcome of patients receiving DAGF. Analysis of both crude mortality rates and adjusted survival rates revealed that there was no statistically significant difference in survival in DAGF patients based on dialysis modality. The results seemed robust as the effect size remained non-significant for both analyses on sequential exclusion of studies.

Importantly, the evidence presented in this review is not from randomized controlled trials but from retrospective cohort studies and therefore should be interpreted with caution. The reason for the selection of PD and HD was not reported by the majority of studies. La Porta et al.^13^ have shown that the probability of being prescribed with PD for DAGF was higher if the patient was treated with PD pre-transplant. Nevertheless, there is a profound tendency to choose HD over PD among graft failure patients. Data from a French registry shows that about 86.7% of graft failure patients choose HD and only 5.1% choose PD.^27^ There could be several reasons for this discrepancy. First, diabetes mellitus is more frequent in post-transplant patients, and given the negative impact of diabetes on PD technique survival physicians may prefer HD.^28^ Secondly, prolonged immunosuppression in transplanted patients can increase the risk of infections particularly peritonitis which is an independent predictor of mortality and technique survival in PD.^29^ However, a meta-analysis has shown that PD initiated after failed allograft is not associated with increased mortality, technique failure, or peritonitis as compared to transplant-naive patients initiating PD.^30^ This indicates that fears of worse outcomes with PD in the transplant failure population are unfounded and they fair similar to transplant-naïve patients. It is also worth noting that the risk of septicemia is high in DAGF patients starting with HD. This is further aggravated by the higher incidence of central venous catheter use in this cohort (2/3^rd^ of the population) and the low prevalence of arteriovenous fistulae/grafts^31^ which increases the risk of mortality.^32^ Thus, it can be noted that both modalities have their limitations in DAGF patients and the choice of dialysis should be individualized considering the patient’s needs.

There are other noteworthy confounders to consider while interpreting the results of our review. First is the timing of dialysis reinitiation which was not adequately reported by the included studies. Limited literature is available on the association between the timing of reinitiation and outcomes of DAGF patients but there may be a tendency for worse survival with early dialysis especially in healthier and younger patients.^33^ Second, the ratio of diabetics was not evenly distributed amongst PD and HD in the study cohorts. Samarasinghe et al.^34^ have shown that diabetic patients with graft failure have a significant survival disadvantage as compared to non-diabetics. Thirdly, there was limited information on the continuation or stoppage of immunosuppressive therapy in the studies. The advantage of maintaining immunosuppressants is that it would maintain residual renal function and prevent graft intolerance syndrome but increase the risk of infection and malignancy.^7^ Ryu et al.^35^ have shown that maintaining immunosuppression significantly decreased the survival of patients with failing grafts as compared to weaning. Thus, despite noting a lack of difference in survival in DAGF patients on PD vs HD, many important questions remain unanswered.

### Limitations

Firstly, despite a large number of transplant failures worldwide, there is limited data published on the choice of dialysis modality. Most of the studies had a small sample size and hence strong conclusions cannot be derived based on available evidence. Second, as mentioned earlier, data was obtained only from retrospective studies. Such data is prone to selection bias which can skew outcomes. Thirdly, only one study used propensity score matching for baseline variables. There was no uniformity between PD and HD cohorts as much of the baseline data was not mentioned by the included studies. Further, not all studies reported adjusted data which reduced the number of studies in the meta-analysis. Lastly, subgroup analysis based on age of patients could not be conducted due to limited number of studies and lack of reporting of separate data by the included studies.

The current results indicate that patients may be offered with both PD and HD after dialysis failure as there may be no difference in survival rates. Hence, other factors like patient preferences, medical suitability, lifestyle, socioeconomic circumstances, and healthcare system resources can dictate the choice of dialysis modality in such patients. Future studies with larger sample size, especially robust multi-centric randomized controlled trials should be conducted to gain further insights on difference in the risk of survival, technique survival, complications, and peritonitis rates in DAGF patients.

## CONCLUSION

Very low quality evidence mostly from retrospective studies shows that dialysis modality may not impact survival rates in patients returning to DAGF.

### Authors’ contributions:

**NZ:** Study design, literature search and manuscript writing.

**NZ and LM** were involved in data collection, data analysis and interpretation. Revision and Revalidation.

All authors have read and approved the final manuscript. They are also responsible for the integrity of the study.
